# Supplementary material for: Caulerpin Mitigates Helicobacter pylori-Induced Inflammation via Formyl Peptide Receptors
Source: Int J Mol Sci. 2021 Dec 5;22(23):13154. doi: 10.3390/ijms222313154 (PMC8658387; doi:10.3390/ijms222313154)
Supplement: Supplementary file 1 [file ijms-22-13154-s001.zip › ijms-1450178-Supplementary information.pdf]

GENE	PRIMER
GAPDH (Glyceraldehyde-3-Phosphate Dehydrogenase)	FW: 5'-CCTCTGACTTCAACAGCGACAC-3'<br>RW: 5'-CACCACCCTGTTGCTGTAGCCA-3'
TNF-α (Tumor necrosis factor alpha)	FW: 5'-CCTGGTATGAGCCCATCTAT-3'<br>RW: 5'-ACAGGGCAATGATCCCAAAGTA-3'
IL8 (Interleukin 8)	FW: 5'-ACTTAGATGTCAGTGCATAAAGAC-3'<br>RW: 5'-TTATGAATTCTCAGCCCTCTTCAA-3'
IL6 (Interleukin 6)	FW: 5'-CCACTCACCTCTTCAGAACG-3'<br>RW: 5'-CATCTTTGGAAGGTTCAGGTTG-3'
SOD2 (Superoxide Dismutase 2)	FW: 5'-CTGATTTGGACAAGCAGCAA-3'<br>RW: 5'-CTGGACAAACCTCAGCCCTA-3'
p53 (Tumor Protein 53)	FW: 5'-AGAGTCTATAGGCCCACCCC-3'<br>RW: 5'-GCTCGACGCTAGGATCTGAC-3'
	**PROBE**
FPR1 (Formyl Peptide Receptor 1)	Hs04235426_s1
FPR2 (Formyl Peptide Receptor 2)	Hs02759175_s1
FPR3 (Formyl Peptide Receptor 3)	Hs00266666_s1
GAPDH (Glyceraldehyde-3-Phosphate Dehydrogenase)	Hs02786624_g1
CCL2 (C-C Motif Chemokine Ligand 2)	Hs00234140_m1
CCL3 (C-C Motif Chemokine Ligand 3)	Hs00234142_m1
CCL5 (C-C Motif Chemokine Ligand 5)	Hs99999048_m1
CCL7 (C-C Motif Chemokine Ligand 7)	Hs00171147_m1
CXCL2 (C-X-C Motif Chemokine Ligand 2)	Hs00601975_m1

Table S1. Primers and probes used for RT-qPCR.

Primary Antibodies	Company	Dilution
Vinculin	Santa Cruz Biotechnology; mouse monoclonal antibody	1:10000
pIkB-α (ser32/36) (phospho Inhibitor of Nuclear Factor Kappa B alpha)	Cell Signaling; rabbit monoclonal antibody	1:250
IkB-α (C-21) (Inhibitor of Nuclear Factor Kappa B)	Santa Cruz biotechnology; mouse monoclonal antibody	1:250
pERK1/2 (th202/tyr204) (phospho Extracellular signal-Regulated Kinases)	Cell Signaling; rabbit monoclonal antibody	1:1000
ERK1/2 (C-9): sc-514302 (Extracellular signal-Regulated Kinases)	Santa Cruz biotechnology; rabbit monoclonal antibody	1:1000
pAkt (ser473) (phospho protein kinase B)	Santa Cruz biotechnology; rabbit monoclonal antibody	1:500
Akt1 (Protein kinase B)	Millipore cat 07416 Lot 28745; mouse monoclonal antibody	1:500
pSTAT3 (tyr705) (phosphor Signal Transducer and Activator of Transcription 3)	Cell Signaling; rabbit monoclonal antibody	1:500
STAT3 (F-2): sc-8019 (Signal Transducer and Activator of Transcription 3)	Santa Cruz biotechnology	1:500
**Secondary Antibodies**	**Company**	**Dilution**
Goat Anti-Rabbit HRP	sc-2027 Santa Cruz Biotechnology	1:5000
Goat Anti-Mouse HRP	sc-2025 Santa Cruz Biotechnology	1:5000

Table S2. Antibodies used for Western Blot analysis.

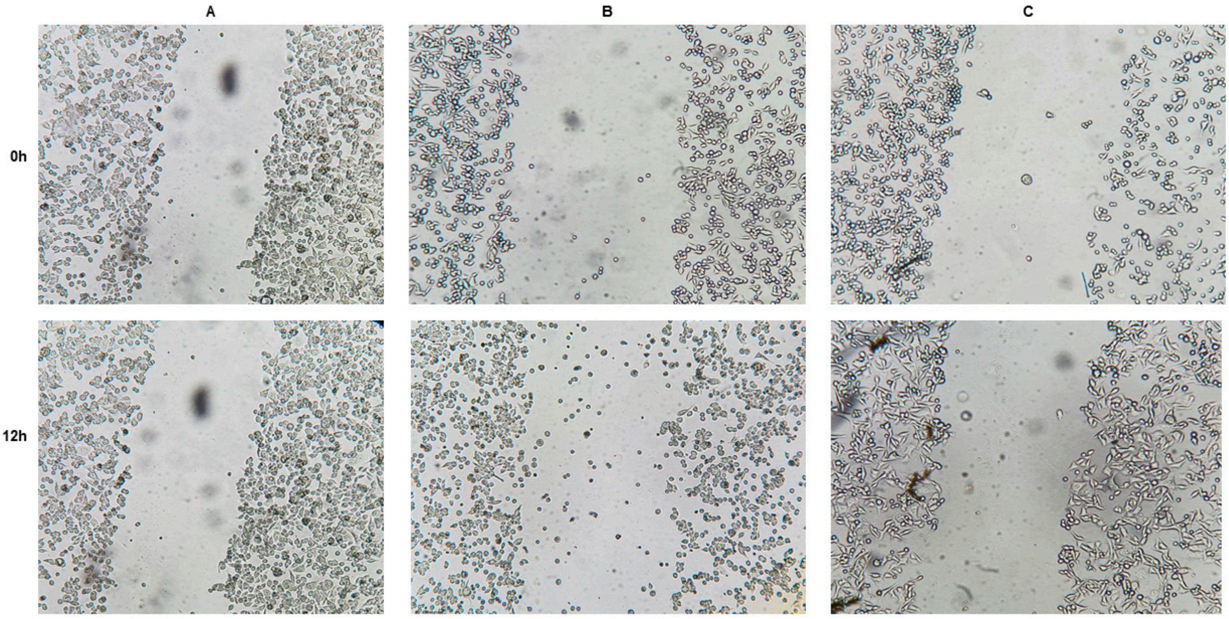

Figure S1**. **Cau prevents Hp(2-20)-induced cell migration. Cell migration by in vitro scratch assay was performed on AGS cells differently treated: (A) untreated; (B) Hp(2-20) 25 μM; (C) Cau 15 μM for 30 minutes and then Hp(2-20) 25 μM. Cells were incubated for 12 hours and pictures were captured before and after cell incubation.

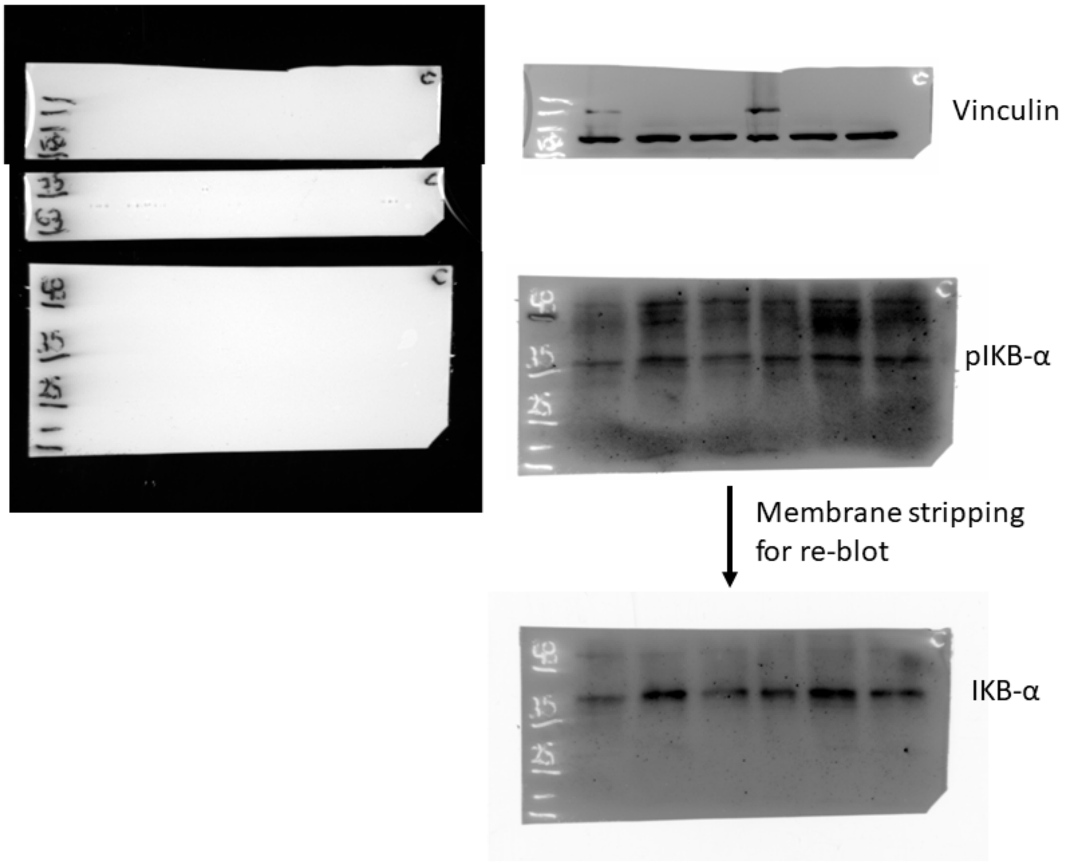

Figure S2. Uncropped and unadjusted Western Blot images corresponding to Figure 2B

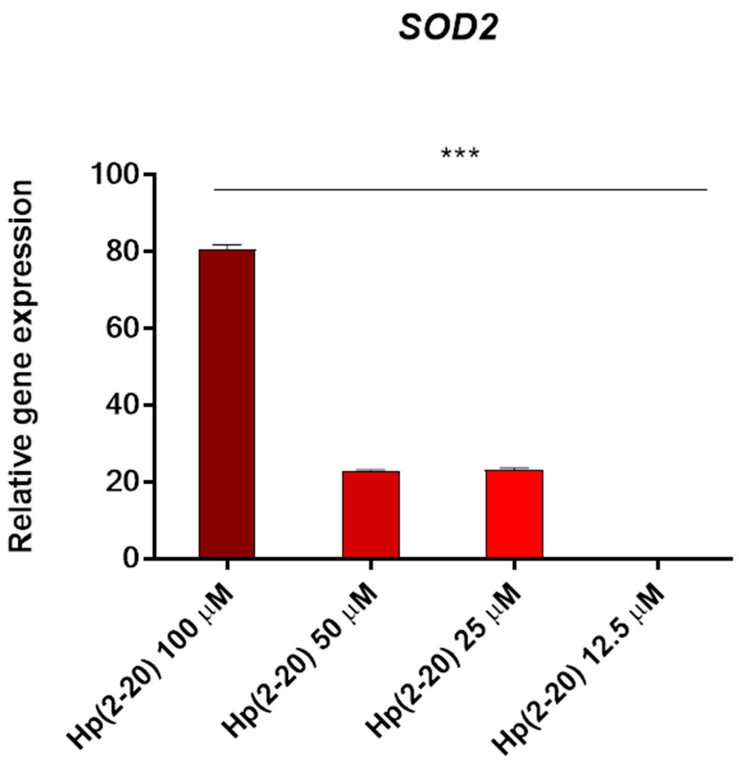

Figure S3*. *Hp(2-20) increases SOD2 gene in a dose-dependent manner. Relative gene expression of *SOD2* by quantitative real-time PCR (qPCR) in cells treated with Hp(2-20) at different concentrations, as indicated. All samples were normalized to GAPDH as reference housekeeping gene. Furthermore, relative gene expression was normalized to basal activity (untreated control), in order to obtain relative fold expression. Graphs report the results of at least three independent experiments, represented as means ± SD. Statistical analysis was performed by GraphPad Prisma software, using one-way ANOVA followed by Bonferroni post-hoc correction. *** $p < 0.0001$.
